# A Practical Sensor-to-Segment Calibration Method for Upper Limb Inertial Motion Capture in a Clinical Setting

**DOI:** 10.1109/JTEHM.2025.3565986

**Published:** 2025-04-30

**Authors:** Mhairi McInnes, Dimitra Blana, Andrew Starkey, Edward K. Chadwick

**Affiliations:** School of EngineeringUniversity of Aberdeen 1019 Aberdeen AB24 3FX U.K.; Aberdeen Centre for Health Data ScienceUniversity of Aberdeen 1019 Aberdeen AB24 3FX U.K.

**Keywords:** Clinical motion analysis, IMU, joint axes estimation, wearable sensors

## Abstract

Inertial sensors have the potential to be a useful clinical tool because they can facilitate human motion capture outside the research setting. A major barrier to the widespread application of inertial motion capture is the lack of accepted calibration methods for ensuring accuracy, in particular the lack of a common convention for calculating the rotational offset of the sensors, known as sensor-to-segment calibration. The purpose of this study was to develop and test a sensor-to-segment calibration method for upper limb motion capture which is practical for clinical applications.We developed a calibration method which depends mainly on the estimation of joint axes from arbitrary elbow motion, and partially on the design of custom attachment mounts to achieve physical alignment. With twenty healthy participants, we used OpenSim’s inertial sensor workflow to calculate joint kinematics, and evaluated the accuracy of the method through comparison with optical motion capture.We found the new calibration method resulted in upper limb kinematics with a median RMS error of 5–8°, and a median correlation coefficient of 0.977–0.987, which was significantly more accurate than a static pose calibration (p-value < 0.001).This work has demonstrated a method of calibration which is practical for clinical applications because it is quick to perform and does not depend on the subject’s ability to perform specific movements, or on the operator’s ability to carefully place sensors.Clinical Impact: The calibration method proposed in this work is a realistic option for the translation of inertial sensor technology into everyday clinical use.

## I. Introduction

Motion capture equipment has been used by clinical researchers to measure the success of medical interventions, deepen our understanding of various diseases, and to improve the design of assistive devices. As new technologies have been developed, the potential to take motion capture outside of the laboratory has become evident, where measurements could be made in various clinical settings, or even at a patient’s home. With flexible, affordable equipment, a patient’s movement could be measured to track the progress of a condition, to influence physiotherapy treatment plans, or for pre and post-operative assessments. With at-home measurement, long-term movement patterns could be recorded, or real-time motion feedback could be used to improve self-guided rehabilitation. Inertial sensors, which use accelerometers, gyroscopes and magnetometers to measure orientation, are an obvious choice for such applications; they are portable, lightweight, wireless, and considerably less expensive than the “gold standard” optical motion capture (OMC) systems used in laboratories. Even though many studies have demonstrated the capabilities of inertial motion capture (IMC) and its potential for accurate measurement of joint angles, the technology has not yet been adopted as a clinical tool. In a recent survey of such studies [1], it was concluded that a major barrier to the uptake of IMC is the lack of common convention for calibrating sensors to their associated bone segments, known as *sensor-to-segment calibration*.

**FIGURE 1.**
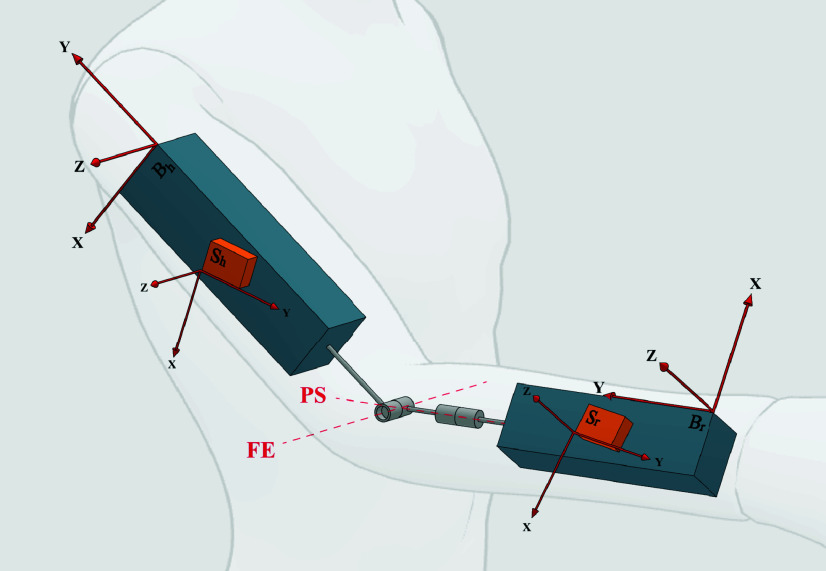
Sensor-to-segment calibration involves the calculation of the rotational offset between the sensor coordinate frame (e.g. $S_{h}$) and the bone segment coordinate frame (e.g. $B_{h}$). To estimate functional joint axes from arbitrary motion, the elbow is modelled as two rigid bodies connected by a 2-DoF joint, where the axes correspond to flexion/extension and pronation/supination movement, where each sensor is rigidly attached to its respective body with an arbitrary orientation.

Sensor-to-segment calibration is the process of calculating the time-invariant rotational offset between each sensor and the associated bone segment (demonstrated in Figure 1), and is usually the first step to IMC. Once this rotational offset is known, the orientation data from the sensors can be used to calculate the orientation of the segments and therefore the joint kinematics, which can be streamed in real-time, or analysed offline. Therefore, the accuracy of the calibration can majorly influence the validity of the calculated joint angles. Various methods have been proposed to perform this sensor-to-segment calibration, each with their own benefits and limitations [1], [2]. The most basic method is a *manual unit alignment*, in which the sensors are carefully placed such that the coordinate frame of each sensor is assumed to be in alignment with the coordinate frame of the associated bone. Accurate placement is difficult to achieve and is therefore very sensitive to the expertise of the operator. To measure the relative orientation of the bone segment coordinate frames more precisely, *anatomical landmark identification* was proposed in [3], where a calibration device is used to locate palpable anatomical landmarks. However, the rigorous process of palpating landmarks accurately is still dependent on operator expertise and could be time-consuming, depending on the number of joint axes to be identified. Other studies have relied on additional systems (such as OMC) to identify these anatomical landmarks [4], [5], however, when the aim is to validate new inertial sensor processing methods, these results do not reflect the effectiveness of inertial sensors as a stand-alone alternative to OMC. The most commonly used sensor-to-segment calibration which does not rely on additional systems is a *static pose* method, in which the subject is asked to perform an initial calibration pose, and the rotational offset of the sensors is calculated under the assumption that the target pose was perfectly executed. In some research studies, with healthy subjects and expert operators, this static pose method has demonstrated good accuracy (<5° RMS error [6]), however, for clinical applications, where patients may have limited control of their movement, achieving the target pose may not be feasible. Furthermore, for at-home use, where patients might need to perform the pose without assistance, an accurate calibration is even less likely. In another method, known as *functional calibration*, subjects are asked to perform specific movements so that the direction of functional joint axes can be estimated from the mean or first principal component of the angular velocity vector, but again, the success of the calibration is dependent on the subject’s ability to carefully isolate each joint axis (e.g., to flex the elbow without pronating or supinating), either actively or passively. When research studies use healthy participants to demonstrate the accuracy of these methods, the same results cannot be expected with affected patients.

To circumvent the issues described above, more recent studies have extended the *functional* approach and developed *model-based* calibration methods, in which arbitrary motion is used to estimate functional axes, based on the constraints of a kinematic model of a joint. Several studies have used a one degree of freedom (DoF) hinge joint model to estimate the primary axis of the knee [7], [8], [9], and a 2-DoF model of the elbow was introduced in [10], where optimisation was used to estimate the flexion/extension (FE) and pronation/supination (PS) axes. In [11], Laidig et al. used a similar algorithm but extended the method for use with six-axis inertial sensors, which do not use magnetometer readings to define a common reference frame, therefore avoiding issues with magnetic interference. From a small cohort of five healthy participants, the optimisation method proposed by Laidig et al. produced consistent estimates of the FE and PS axes. However, to calculate the elbow joint kinematics, an initial reference pose was still used to set the “zero-point” of the FE and PS joint angles. Furthermore, the study was limited to the elbow joint, and did not investigate whether the estimate of the FE axis could be used to calibrate shoulder joint angles.

In comparison to other methods, model-based calibration has been shown to measure elbow joint kinematics with high levels of accuracy (2–4° RMS error [6]), and would be practical for clinical application because, theoretically, it is quick and easy to perform and does not depend on the subject to execute accurate poses or movements. In a survey of sensor-to-segment calibration methods [1], it was concluded that model-based methods are promising, but require significant further validation.

In terms of upper limb motion analysis, another commonly identified barrier is access to software [12]. In the field of biomechanics, OpenSim [13] is a widely used open-source software, and a new IMC tool has recently been released, called OpenSense [4]. The OpenSense tool allows the user to choose a musculoskeletal model, define the orientation of virtual inertial sensors in the model using a static pose sensor-to-segment calibration, and then use recorded inertial sensor data to calculate joint kinematics. The open-source nature of this software and the well-established community of international users makes it a great facilitator for the development of common conventions for IMC, but the use of a static pose calibration makes it impractical for clinical applications.

The purpose of this research was to develop and test a new sensor-to-segment calibration method for both shoulder and elbow joint kinematics which, in comparison to existing methods, is less reliant on the abilities of the subject or operator. The method uses two concepts to achieve an accurate calibration:

1) Estimating the elbow FE axis and forearm PS axis from arbitrary elbow motion using a model-based method similar to that proposed in [11].
2) Relying on the design of the attachment mounts to achieve self-alignment of some sensor axes with their associated bone axes.

This paper describes the proposed calibration method, then evaluates the accuracy of the method through concurrent measurement with optical motion capture (OMC). OpenSense is used to calculate joint kinematics, and the new calibration method is compared with the built-in static pose method.

## II. Methods

### A. Proposed Calibration Method

**FIGURE 2.**
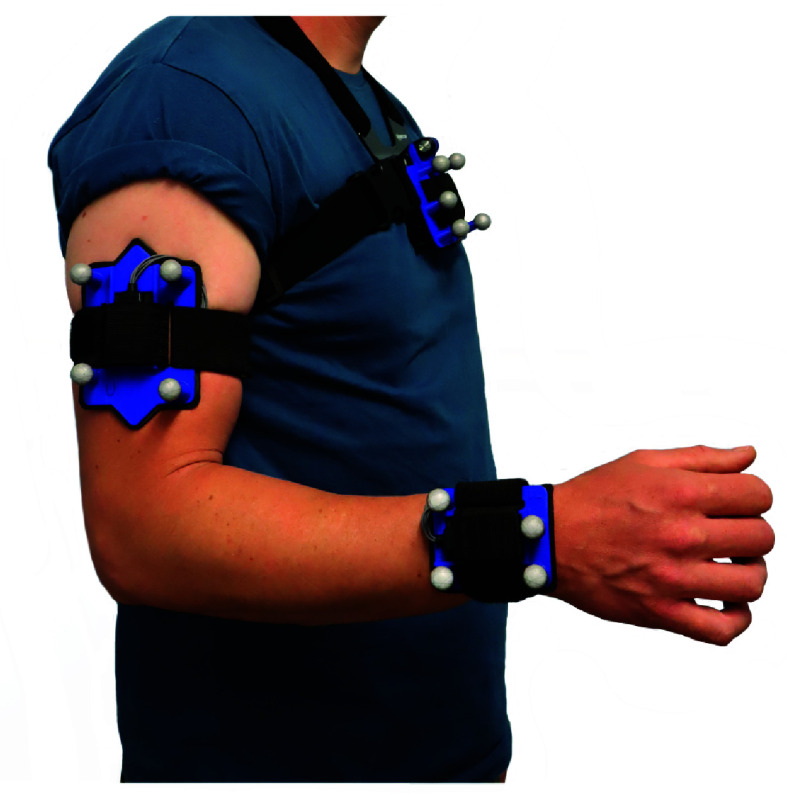
Experimental set-up, where inertial sensors and reflective marker clusters were attached to the sternum, upper arm and forearm. A pointer, mounted with markers, was used to define anatomical landmarks relative to the marker clusters.

The following method is intended for measuring elbow and shoulder (humero-thoracic) angles with quaternion orientation data from inertial sensors attached to the sternum (thorax sensor), upper arm (humerus sensor), and forearm (radius sensor), as in Figure 2. To calculate joint angles, the sensor-to-segment rotational offset, $q_{B}^{S}$, must be calculated for each sensor-bone pair. For the humerus and radius sensors, the proposed method calculates $q_{B}^{S}$ by first estimating the direction of the functional joint axes, FE and PS, then by assuming that one of the sensor axes is in alignment with one of the bone axes. A short period of elbow movement is required to estimate the functional joint axes, and the impact of the type of movement performed is discussed in Section III. The assumption that one of the sensor axes is in alignment is dependent on the design of the attachment mount, which is discussed in Section II-A3. The thorax sensor alone is calibrated using a static pose, where it is assumed that the subject can achieve an upright posture.

#### 1) Estimating Joint Axes

The functional joint axes were estimated using an adapted version of the algorithm proposed by Laidig et al. [11], which models the elbow as a 2-DoF joint, as depicted in Figure 1. The objective of the algorithm is to estimate the direction of the flexion/extension axis, $\boldsymbol {j}_{FE}$, relative to the humerus sensor frame, $S_{h}$, and the pronation/supination axis, $\boldsymbol {j}_{PS}$, relative to the radius sensor frame, $S_{r}$, based on inertial sensor data recorded during arbitrary elbow motion. Laidig’s method is based on the addition theorem for angular velocities, which defines the relationship between the angular velocity of two bodies connected by a 2-DoF joint as

\begin{equation*} [{\boldsymbol {\omega }}_{S_{r}}]_{\varepsilon }- [{\boldsymbol {\omega }}_{S_{h}}]_{\varepsilon }= \omega _{j_{FE}}[\boldsymbol {j}_{FE}]_{\varepsilon }+ \omega _{j_{PS}}[\boldsymbol {j}_{PS}]_{\varepsilon }. \tag {1}\end{equation*}

The vectors ${\boldsymbol {\omega }}_{S_{h}}$ and ${\boldsymbol {\omega }}_{S_{r}}$ are the angular velocities of the humerus and radius sensors, $\boldsymbol {j}_{FE}$ and $\boldsymbol {j}_{PS}$ are the axes which define the 2-DoF joint, and the scalars, $\omega _{j_{FE}}$ and $\omega _{j_{PS}}$ are rotation rates around the respective joint axes. The $\varepsilon $ notation denotes that these vectors are expressed in a common reference frame. From (1), Laidig et al. derived the constraint,

\begin{equation*} ([{\boldsymbol {\omega }}_{S_{r}}]_{\varepsilon }- [{\boldsymbol {\omega }}_{S_{h}}]_{\varepsilon }) \cdot \frac {[\boldsymbol {j}_{FE}]_{\varepsilon }\times [\boldsymbol {j}_{PS}]_{\varepsilon }}{\lVert [\boldsymbol {j}_{FE}]_{\varepsilon }\times [\boldsymbol {j}_{PS}]_{\varepsilon }\rVert } = 0 \tag {2}\end{equation*}

which, for a “perfect” 2-DoF joint, should be fulfilled for any sampling instant. Although the humerus and radius sensors are not connected by a “perfect” 2-DoF joint (the elbow itself is not a perfect mechanical joint and the sensors are also subject to soft tissue artefacts (STAs) because they are not rigidly connected to the bone [14]) this constraint can be posed as the cost function of a least-squares optimisation problem, for a given set of data samples.

Laidig further extended the problem to consider a case in which the orientation measurements available are from six-axis inertial sensors, which do not use magnetometer measurements and therefore have some unknown heading-offset between the sensor reference frames, $\varepsilon _{S_{h}}$ and $\varepsilon _{S_{r}}$. Although this proposal has great potential for avoiding issues with magnetic interference, preliminary testing of the algorithm found it to be very sensitive to the type of movement used for the calibration. As mentioned by the authors in their own discussions, if the upper arm is not moved sufficiently during the calibration period, the optimisation fails to find a plausible solution for the FE axis. Therefore, we adapted Laidig’s algorithm for use with nine-axis sensors, which *do* use magnetometer readings, and therefore share a common reference frame with no heading offset.

With this adaptation, the optimisation problem follows the steps described in [11], with a simplified error function. For each sampling instant, $t_{k}$, the error is defined by the relative angular velocity of the sensors, and the estimates for $\boldsymbol {j}_{FE}$ and $\boldsymbol {j}_{PS}$,

\begin{equation*} e(t_{k}):= \boldsymbol {\omega }_{rel}(t_{k}) \cdot \frac {[\boldsymbol {j}_{FE}]_{\varepsilon }\times [\boldsymbol {j}_{PS}]_{\varepsilon }} {\lVert [\boldsymbol {j}_{FE}]_{\varepsilon }\times [\boldsymbol {j}_{PS}]_{\varepsilon }\rVert } \tag {3}\end{equation*}

For a given set of samples, M, we find a solution for the vectors $\boldsymbol {j}_{FE}$ and $\boldsymbol {j}_{PS}$ which minimises the sum of the squared errors using a Gauss-Newton algorithm. The gradients of the cost function are calculated as in [11], except the derivatives with respect to the heading offset are not included, because it is no longer a dependent variable. For inertial sensors with on-chip sensor fusion algorithms, which may not report raw angular velocity data, the angular velocity can be calculated from the reported orientation data. For any sensor, *S*, the rotation quaternion from sample $t_{k-1}$ to $t_{k}$, expressed in the global frame, is calculated as,

\begin{equation*} \left [{{\boldsymbol {q}^{S(t_{k})}_{S(t_{k-1})}}}\right ]_{\varepsilon }= \boldsymbol {q}^{S(t_{k})}_{\varepsilon } \otimes \left ({{\boldsymbol {q}^{S(t_{k-1})}_{\varepsilon }}}\right )^{-1}, \tag {4}\end{equation*}

and the angular velocity between each consecutive sample is calculated from the angle-axis $(\alpha {\text{@}} \boldsymbol {v})$ representation of $\left [{{\boldsymbol {q}^{S(t_{k})}_{S(t_{k-1})}}}\right]_{\varepsilon } $,

\begin{equation*} \boldsymbol {\omega } = \frac {\alpha \boldsymbol {v}}{\Delta t}. \tag {5}\end{equation*}

where $\Delta t$ is the period between samples $t_{k-1}$ and $t_{k}$.

#### 2) Refining the Calibration

The output of the optimisation step is an estimate of the FE and PS axes, expressed in the local coordinate frames of the humerus and radius sensors respectively: $[\boldsymbol {j}_{FE}]_{S_{h}}$ and $[\boldsymbol {j}_{PS}]_{S_{r}}$. Given a generic skeletal model of the upper limb, which defines the kinematic constraints of the connected bones (details given in Section II-B2), we can define the direction of the same functional axes, relative to the coordinate frames of the bone segments: $[\boldsymbol {j}_{FE}]_{B_{h}}$ and $[\boldsymbol {j}_{PS}]_{B_{r}}$. Therefore, the rotational offset between the sensor and the bone segment should be found such that the estimated axis in the sensor frame is aligned with the axis in the bone frame, i.e., the following equation should be satisfied:

\begin{equation*} [\boldsymbol {j}]_{B} = \boldsymbol {q}_{B}^{S} \otimes [\boldsymbol {j}]_{S} \otimes ({\boldsymbol {q}_{B}^{S}})^{-1} \tag {6}\end{equation*}

where $\boldsymbol {j}$ is either $\boldsymbol {j}_{FE}$ or $\boldsymbol {j}_{PS}$. However, given the vectors $[\boldsymbol {j}]_{B}$ and $[\boldsymbol {j}]_{S}$, (6) does not give a unique solution for $q_{B}^{S}$ - aligning a single pair of vectors does not fully constrain the rotational offset between two coordinate frames. There is still an undefined degree of freedom where the sensor frame could be rotated by any amount around the vectors which have been aligned. To define this final degree of freedom, we rely on the design of the attachment mounts to align one of the sensor axes with one of the bone axes, which gives a second vector pair. For the humerus, it is assumed that the y-axis (long-axis) of the sensor is aligned with the negative y-axis of the humerus bone, giving the vector pair,

\begin{equation*} \begin{array}{ccc} [\boldsymbol {v}_{y}]_{S} = \begin{bmatrix} 0 & 1 & 0 \end{bmatrix} & \text {and} & [\boldsymbol {v}_{y}]_{B} = \begin{bmatrix} 0 & -1 & 0 \end{bmatrix}. \end{array} \tag {7}\end{equation*}

And for the radius, it is assumed that the x-axis of the sensor (along the width) is aligned with the x-axis of the radius, giving the vector pair,

\begin{equation*} \begin{array}{ccc} [\boldsymbol {v}_{x}]_{S} = \begin{bmatrix} 1 & 0 & 0 \end{bmatrix} & \text {and} & [\boldsymbol {v}_{x}]_{B} = \begin{bmatrix} -1 & 0 & 0 \end{bmatrix}. \end{array} \tag {8}\end{equation*}

Computing the rotation between two frames, *B* and *S*, given pairs of observed vectors, ( $[\boldsymbol {j}]_{B}$, $[\boldsymbol {j}]_{S}$) and ( $[\boldsymbol {v}]_{B}$, $[\boldsymbol {v}]_{S}$), is known as Wahba’s problem and can be solved with singular value decomposition [15] to find an optimal solution for $q_{B}^{S}$ in a least-squares sense. A weighting is applied to each vector pair so that the joint axes estimates are heavily prioritised ( $\text {weight}=1$) and the manual unit alignment is only used to constrain the undefined degree of freedom ( $\text {weight}=0.01$).

The output of this computational step is, for both the humerus and radius, a rotational offset $q_{B}^{S}$ which defines the sensor-to-segment calibration. The solution is primarily based on the estimation of joint axes from arbitrary motion, and secondarily based on the assumption of alignment between an axis of the sensor frame with an axis of the bone frame.

#### 3) Attachment Mount Design

The success of the calibration algorithm described above relies partially on the accurate alignment of the humerus and radius sensors with their associated bone segments. However, existing calibration methods which rely on manual alignment have been dismissed as impractical because they depend on the intricate placement of the sensors. Therefore, the humerus and forearm attachment mounts were designed to maximise alignment, without depending on careful placement.

**FIGURE 3.**
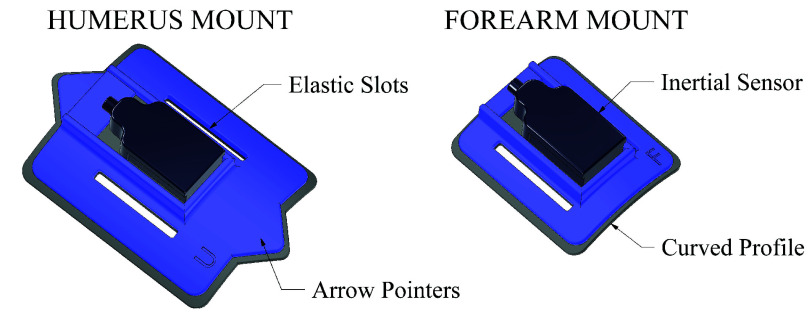
Custom 3D-printed attachment mounts, designed to maximise alignment between the sensor and the underlying bone segment. The curved profile was chosen to best fit a range of body sizes and wide elastic straps were intended to reduce twisting. Raised cones for reflective marker attachment were also included for experimental validation.

Both mounts were 3D printed in a rigid PLA plastic, with a rectangular recess for the sensors, shown in Figure 3 (3D CAD files and manufacture instructions are shared in the supplementary material). Wide elastic straps (40 mm) were used to secure the mounts and the curved profile was intended to hug the length of the limb and minimise twisting. The radius of the curve was chosen to best fit the shape of the limb, whilst accommodating for a range of body types. The curvature of the forearm mount is less pronounced to match the flatter surface of the intended location: at the distal end of the forearm, just proximal from the radial and ulnar styloids (as in Figure 2). This location was chosen to best capture axial rotation of the limb, and because the muscle and adipose tissue below the skin is usually minimal in this area, regardless of the individual’s body composition. The humerus mount was elongated to minimise twisting and is intended to be secured mid-way down the upper arm, facing laterally when in the neutral pose. Triangular arrows were also incorporated into the humerus mount as visual markers for the operator to check for alignment. Lastly, reflective markers were affixed, slightly projected from the face of the mounts (seen in Figure 2) which was only necessary for concurrent validation.

It should be noted that the success of the calibration does not depend on all three axes of the sensor frame aligning with the bone frame, but only the y-axis of the humerus sensor, and the x-axis of the radius sensor.

### B. Experimental Evaluation

An experimental trial was carried out to evaluate the accuracy of the proposed calibration method. Subsidiary objectives were to quantify the success of the joint axes estimation algorithm, and to measure the reliability of the attachment mounts to self-align on a range of body types.

#### 1) Experimental Set-up

To meet the experimental objectives, informed consent was obtained from twenty healthy participants (10F, 10M; $40.0~\pm ~12.1$ yrs.; $172.0~\pm ~11.4$ cm; $77.0~\pm ~18.3$ kg) who were asked to perform a selection of simple movements whilst their trunk and right arm motion was measured by inertial sensors and an optical motion capture system (OMC) simultaneously. Using the custom attachment mounts, an inertial sensor and a cluster of retroreflective markers were attached to the sternum, upper arm, and forearm, as in Figure 2. The 3D position of the retroreflective markers was measured at 120 Hz by an OMC system (OptiTrack, NaturalPoint, Corvallis, OR, USA). The inertial sensors (Delsys Trigno sensors (Delsys Inc., Boston, MA, USA)) used an on-chip sensor fusion algorithm to calculate quaternion orientation data, which was transmitted at a sampling frequency of 74 Hz to a receiver via Bluetooth. Both position and orientation data were time-synchronised within The MotionMonitor software (Innovative Sports Training, Chicago, IL, USA) where a low pass fast Fourier transform filter was applied (5 Hz cut-off frequency), any short gaps (< 0.5 s) in the marker position measurements were repaired by linear interpolation, and all data was re-sampled to 100 Hz. A pointer, mounted with markers, was used to define anatomical landmarks relative to the marker clusters (the CAST method [16]) and the position of the landmarks was also recorded during movement. (Anatomical landmarks: C7, T8, IJ, PX, AA, EM, EL, RS, US.)

The operator ensured the mounts were donned as described in Section II-A3, then tightened the elastic until they felt secure, allowing the mounts to “seat” automatically. Participants were asked to perform the neutral pose given a simple set of instructions (“upright posture, arm by your side, elbow straight, thumb pointing forward”) and an example image of the pose. The operator then gave verbal and physical feedback to improve the pose wherever they saw errors, giving us a “self-executed” and “assisted” version of the pose. Participants were then asked to perform two movement trials. The first trial, “isolated movements”, involved five repetitions of movement around each joint axis of the elbow and shoulder: shoulder flexion/extension, shoulder abduction/adduction, shoulder internal/external rotation, elbow flexion/extension, pronation/supination, at a slow speed (approximately 1 Hz). The second trial, “functional movements”, involved a series of typical activities of daily living, chosen to move the upper limb through the range of reachable workspace, including ‘drinking from a cup’, ‘opening a high cupboard’, and ‘pouring from a kettle’, all performed at a self-selected speed. The average range of motion (ROM) of each joint angle during these tasks can be found in Appendix B, supplementary material.

#### 2) OMC Reference Kinematics

From the recorded marker position data, reference joint kinematics were calculated using OpenSim’s marker-based inverse kinematics tool. The skeletal model (henceforth known as the OMC model) was adapted from the “Dynamic Arm Simulator” model [17] and consisted of a thorax, clavicle, scapula, humerus, radius, and ulna. The thorax was free to translate and rotate relative to the ground, and the thorax, clavicle, scapula and humerus were connected by a series of 3-DoF joints, allowing for realistic translation of the shoulder girdle. Elbow flexion was defined by a 1-DoF joint between the humerus and ulna, and pronation/supination by a 1-DoF joint between the ulna and radius. The position of anatomical markers were already defined in the model, relative to each associated bone segment. The direction of the FE and PS joint axes defined in this model were used as the reference vectors for the calibration step described in Section II-A2. Further model details are given in Appendix A, supplementary material.

For each individual, the dimensions of the OMC model bone segments were scaled based on the distance between proximal and distal anatomical landmarks, and the position of the anatomical landmarks in the model were adjusted to better match the individual based on a single time sample. With the scaled model, OpenSim’s inverse kinematics tool was used to calculate reference joint kinematics. The time-varying joint angles extracted from the OMC kinematic model were elbow flexion/extension, forearm pronation/supination, and thoraco-humeral shoulder abduction, flexion, and rotation. To calculate the joint angles of the shoulder, projected vectors were used instead of Euler angles, which are sensitive to gimbal lock. Abduction/adduction and flexion/extension were defined as the direction of the humerus long axis projected onto the thorax frontal, and sagittal planes, respectively. Internal/external rotation was defined by the projection of the humerus x-axis on the thorax transverse plane.

#### 3) Inertial Sensor Kinematics

From the recorded sensor orientation data, OpenSim’s IMU inverse kinematics tool was used to calculate joint kinematics. The same model described in Section II-B2 was used, except the sternoclavicular and acromioclavicular joints were locked because there were no sensors attached to the scapula or clavicle. Therefore, shoulder motion was allowed only by the 3-DoF glenohumeral joint. A virtual IMU was associated with the thorax, humerus and radius segments, each with a fixed rotational offset, defined in the coordinate frame of the associated bone segment. Before running the inverse kinematics tool, the rotational offset of each virtual IMU was defined to create a calibrated model by applying one of two calibration methods: the static pose method built-in to the OpenSense workflow, or the new calibration method, applied through the OpenSim API through python scripting. To apply the new calibration, the self-executed version of the calibration pose was used to calibrate the thorax sensor, then a period of movement was specified as the input to the joint axes estimation algorithm, the results of which were used to calibrate the humerus and radius sensors.

To assess the efficacy of the method with different types of calibration movement, periods of data from different movement trials were used as the input to the calibration. From the “isolated” movement trial, two different periods were defined: “5 reps”, where the participant performed five repetitions of FE and then five repetitions of PS, and “1 rep”, where only one repetition of FE and PS were performed. From the “functional” movements trial, three different periods of data were used: “pouring a kettle”, “drinking from a cup”, and “both”, which encompassed both tasks.

For each individual, the steps above produced a different calibrated model depending on the method and data used. For each calibrated model, the OpenSense IMU inverse kinematics tool was then used to compute joint kinematics. This tool solves a multi-body optimisation problem where, on a frame-by-frame basis, the model is posed such that the error between the virtual sensor orientation and the orientation of the input sensor data is minimised in a least-squares sense. Weightings can also be applied to each sensor, based on pre-defined knowledge of the system or specific confidences in each sensor. Based on preliminary testing, the sensor attached to the humerus was most susceptible to soft tissue artefacts, therefore, the humerus sensor was down-weighted by a factor of ten, in comparison to the other two sensors (the weight of the thorax, humerus and radius sensors was 1.0, 0.1, and 1.0, respectively). All inverse kinematic computations were solved with these relative weightings.

#### 4) Evaluation Metrics

The accuracy of the inertial sensor kinematics was assessed using the OMC-derived kinematics as a “ground-truth” reference. For each joint angle, the RMS error and Pearson’s correlation coefficient were calculated and the median and interquartile range across all subjects is reported in the results.

To evaluate the success of the joint axes estimation algorithm, we compared the direction of the estimated joint axes with the joint axes of the calibrated OMC model, calculating the angle between the two 3D vectors. It should be noted that this comparison is not an exact measure of the accuracy of the estimated axes, because the joint axes in the OMC model are not perfect measures of each individual’s functional joint axes. The joint axes in the OMC model were originally defined from cadaveric measurements of a single individual, therefore, even for a calibrated model, the direction of these axes may not match the subject’s functional axes of movement. For example, the direction of the elbow flexion axis relative to the humerus will vary depending on the individual’s carrying angle, which may not match the OMC model’s. Despite this, these axes can still be used for comparison to assess whether the algorithm has output a sensible estimate. The estimates of the FE and PS axes were deemed “plausible” if they differed by less than 20° from the reference vector.

The success of the attachment mounts was measured using the coordinate frames defined by each cluster of reflective markers. On each attachment mount, the placement of the reflective markers was chosen such that an orthogonal coordinate frame could be built from their relative positions, which was aligned with the casing of the sensor, therefore representing “perfect” inertial sensor frames. The orientation of the “perfect” sensors with respect to the bone coordinate frames was calculated from the OMC measurements, therefore, the success of the sensor-to-segment alignment could be quantified, without incorporating error from the real inertial sensors. The alignment error was defined as the angle between the two axes which should have been aligned, for example, the angle between the humerus sensor’s y-axis, and the humerus bone’s negative y-axis.

## III. Results

### A. Joint Axes Estimation

**FIGURE 4.**
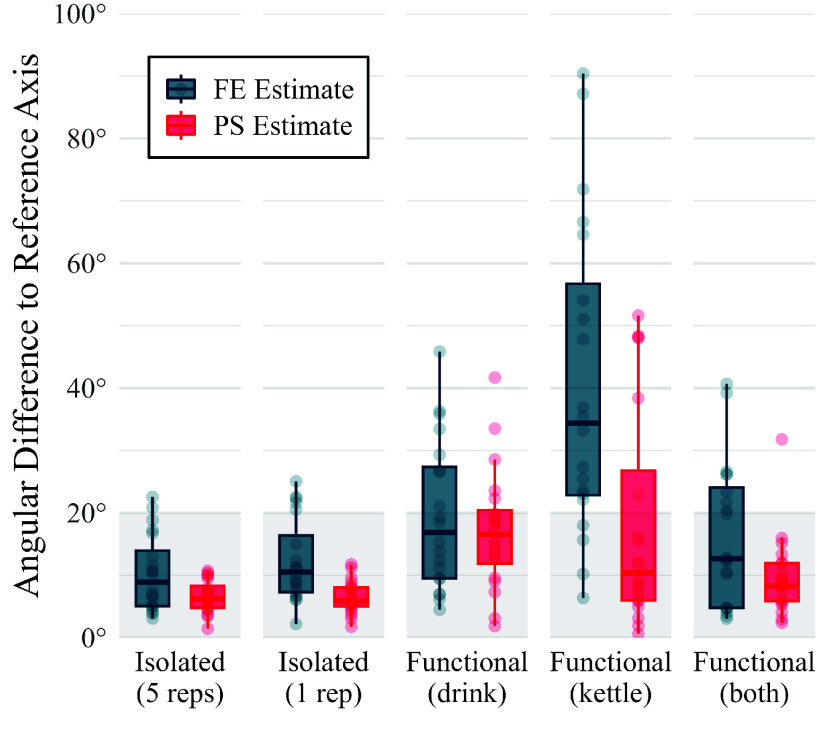
Evaluation of the optimisation algorithm used to estimate joint axes, FE and PS, given different periods of movement data, from either “isolated” or “functional” tasks. Axis estimates were deemed plausible if the angular difference to the reference axis was less than 20°.

With inertial sensor data from the “isolated” movements trial, the adapted optimisation algorithm estimated a plausible PS axis for all subjects, shown in Figure 4. Plausible results were still achieved when only 1 repetition of the movement was performed, showing that the PS axis could be estimated very quickly (5–10 s) with minimal data samples. For all tests, the FE estimate was further from the expected reference vector than PS, perhaps due to the larger soft-tissue-artefacts (STAs) experienced by the humerus sensor. With 5 repetitions, FE estimates were plausible for all but two subjects, and with only 1 repetition, FE estimates were plausible for all but five subjects, and these five were still within 25° of the reference vector. Detailed results can be found in Appendix B, supplementary material.

When the movement period was defined by a “functional” task, the estimated axes were less similar to the expected reference vectors, demonstrated in Figure 4. This may be attributed to the larger range and speed of these movements, which will increase STAs, causing the sensors’ relative motion to deviate further from the 2-DoF model. For example, the average ROM in shoulder flexion was only 18° during 5 reps of the “isolated” movement, but was 64° during the “functional” tasks. This result shows that the success of the algorithm was somewhat sensitive to the type of movement data used.

An interesting observation is that the results from both the “kettle” and the “drinking” task were particularly unreliable, but when the combination of both tasks were used, both the FE and PS axes were closer to the expected reference vectors. Further investigation showed that the range in either PS and FE motion may not have been sufficient during the individual tasks. During these periods of movement, the average ROM in the FE and PS joint angles was as follows: 52° and 88° during the “kettle” task, 107° and 54° during the “drinking” task, and 93° and 131° during the period including both tasks. This suggests that the success of the optimisation might be dependent on the range, or “richness” of the calibration movement, and that increasing the *amount* of input movement data may improve the estimation if the movement in a short period was not sufficiently rich.

### B. Attachment Mount Alignment

**FIGURE 5.**
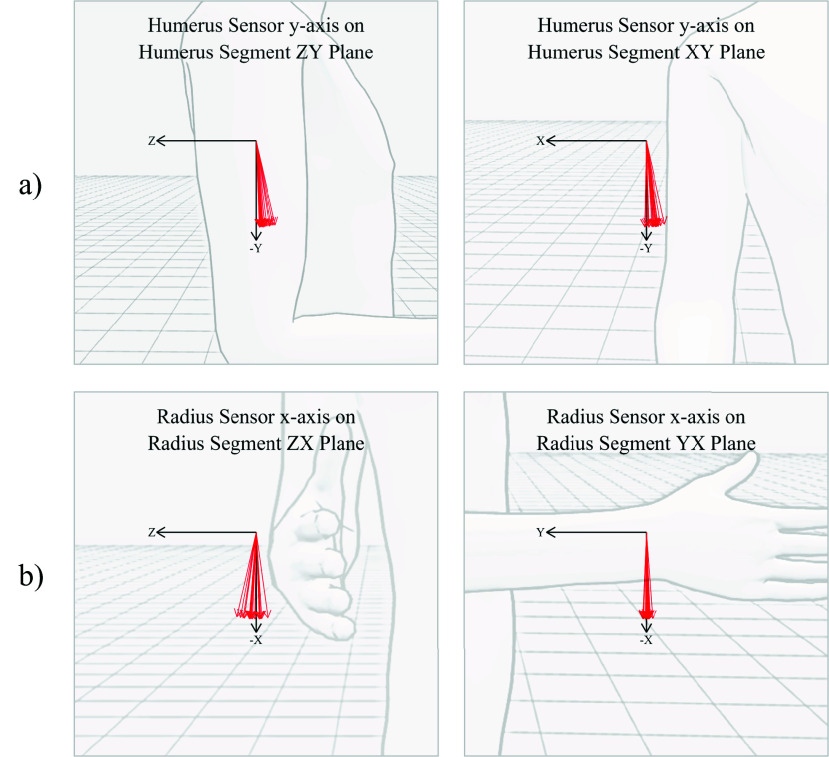
Alignment of the humerus sensor’s y-axis (a) and radius sensor’s x-axis (b) projected onto two planes of the bone’s coordinate frame, for all 20 subjects. For the humerus sensor, the average error on the ZY (shoulder flexion) plane was 5.7° (3.2°) and on the XY (abduction) plane was 5.2° (3.4°). For the radius sensor, the average error on the ZX plane was 4.7° (5.1°) and on the YX plane was 1.3° (1.6°).

The average error in alignment between the humerus sensor’s y-axis and the humerus bone’s negative y-axis was 8.4° (3.0°) (mean, SD). To consider how this misalignment might affect the accuracy of the measured joint angles, the error can be visualised on two separate planes, as in Figure 5. In both planes, a slight bias can be seen in the humerus mount’s alignment, where the top of the sensor is often tilted posteriorly and laterally, suggesting that the underlying musculature can affect how the mount tends to seat, relative to the humerus. For example, the deltoids bulging at the top of the humerus may be causing the top of the mount to tilt laterally.

For the radius sensor, the average misalignment between the sensor’s x-axis and the radius bone’s negative x-axis was 5.1° (3.0°). When visualised on each plane, there is very little misalignment on the XY (elbow flexion) plane, suggesting that the curve of the mount was effectively aligning the long axis of the sensor. However, there was more variation in error on the ZX (pronation) plane, showing that the placement of the mount around the long axis of the forearm was less consistent. Detailed results can be found in Appendix B, supplementary material.

### C. Accuracy of Proposed Calibration Method

**FIGURE 6.**
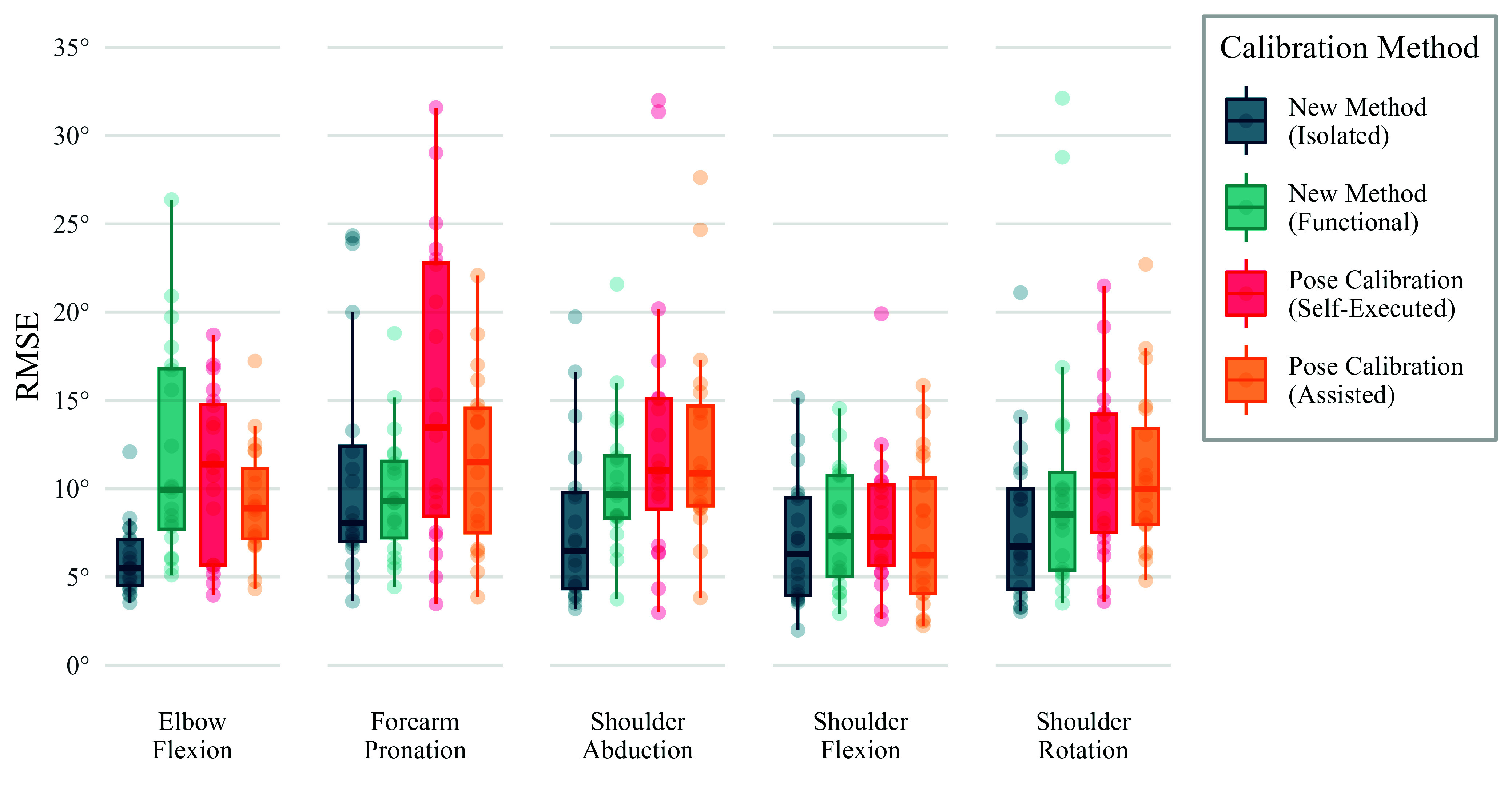
RMS error in the IMC joint kinematics from the “isolated” movements trial using different methods to achieve the sensor-to-segment calibration. With the proposed method, movement from either the “isolated” or “functional” movement trial was used to estimate joint axes, and with the static pose method, either an self-executed or assisted pose was used.

From the “isolated” movements trial, Figure 6 shows the RMS errors of the calculated joint kinematics, comparing the different methods which were used to create each individual’s calibrated model. Using the proposed calibration method, with just one rep of FE and PS movement to calibrate the joint axes, the median RMSE across all subjects was 5–8°, depending on the joint angle, and the median correlation coefficient was over 0.97, for all joint angles. In fact, for all subjects bar one (P010, shoulder abduction, R =0.88) the correlation coefficient was over 0.90 for all joint angles. Elbow flexion/extension was the most accurate and reliable joint angle to be tracked, with an average RMSE of 5.5° (2.6°) (median, IQR), and pronation/supination was the least, with an average RMSE of 8.1° (5.4°). Detailed results can be found in Appendix B, supplementary material.

Using data from the “functional” movement (both tasks) to calibrate the subjects resulted in higher RMSEs and lower correlation coefficients, as expected from the initial testing of the optimisation algorithm. In comparison to the calibration using “isolated” movement, the average error in elbow flexion and shoulder abduction angles was higher, and two subjects had particularly large errors in the shoulder rotation angle (P018: 28.8° and P014: 32.1°). However, the median RMSE over all subjects was still less than 10° for all joint angles.

**FIGURE 7.**
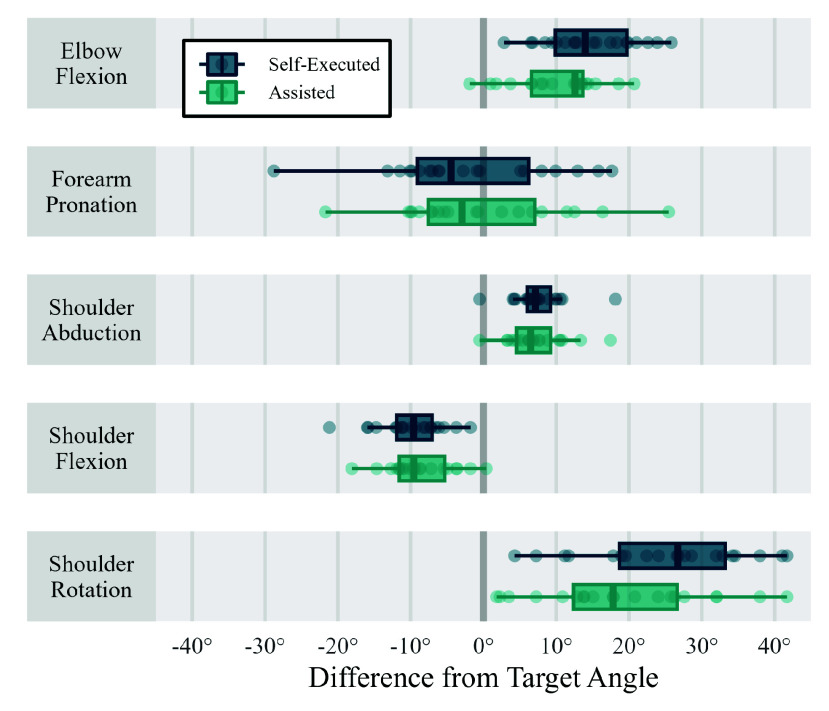
Joint angles achieved during the static calibration pose across all 20 subjects, showing the error relative to the target angle (0°). Very little improvement can be seen between the self-executed and assisted poses.

The other two calibration methods presented in Figure 6 are static pose calibrations, where in one instance, the subjects were asked to perform the pose with some basic instructions, and in the other, some assistance was provided by the operator to improve the execution of the pose. From both methods, the median joint angle RMSEs were 7–14°, and providing assistance to the participant resulted in very little reduction in kinematic errors. The source of these errors can be explained by analysing how accurately the subjects were able to achieve the target pose, calculated from the OMC measurements, and shown in Figure 7. A large variation is evident in some joint angles, such as pronation and shoulder rotation, and others have less variation but a clear offset, such as shoulder abduction, where subjects tended to hold their arm in slight abduction (median: 7.1°), but achieved a similar angle with relative consistency (IQR: 3.2°). Additionally, in many instances, we found that subjects were unable to achieve 0° elbow flexion due to stiffness. Finally, we found that providing assistance only marginally improved the accuracy of the pose. Comparing calibration methods, the proposed method (using just one rep of FE and PS) resulted in lower average kinematic errors than either of the static pose calibrations. A Wilcox signed rank test was carried out and the results indicated a statistically significant difference, with the proposed method showing lower errors than either of the static pose methods (p-values <0.001).

## IV. Discussion

In this study, we evaluated a sensor-to-segment calibration method for upper limb inertial motion capture which is quick to perform and does not depend on careful measurements or precise motions or poses. With a calibration protocol which involves donning the mounts by tightening elastic straps, holding a single static pose to calibrate the thorax, and a movement of just one repetition of elbow flexion/extension and one repetition of pronation/supination (which took approximately 5–10 s), the proposed method measured shoulder and elbow joint angles with median RMSEs of 5–8°, depending on the joint angle, and the median correlation coefficient was over 0.97, for all joint angles. The method relied mainly on the axis estimation algorithm adapted from [11], which performed well given only a short period of data but was dependent on sufficient motion around both the FE and PS axes. The method also relied partially on the alignment achieved by the design of the attachment mounts, which worked consistently for both humerus and forearm mounts, with the largest variation in misalignment present on the pronation/supination plane (4.8° (5.1°)).

The proposed calibration method can be evaluated against existing methods in terms of clinical feasibility, and by comparing joint kinematic errors with those from other research studies which have focused on upper limb calibration and have also used optical motion capture as a concurrent ground truth reference.

Firstly, *model-based* calibration methods, like the joint axis estimation algorithm used in this study, are designed to estimate the direction of joint axes from “arbitrary motion” (e.g., simultaneous FE and PS movement), offering a potential advantage over existing *functional* calibration methods, which rely on constrained motion to generate angular velocity vectors representative of the functional joint axes. In [18], de Vries et al. evaluated the accuracy and repeatability of a *functional* calibration method for the upper limb on five healthy subjects, estimating joint axes directly from angular velocity vectors, averaged over a period of constrained, uni-axial movements. In comparison to the OMC segment axes defined by bony landmarks, they found an average misalignment of 4.0° in the PS axis, and 6.0° in the FE axis, and found low variation in the estimated axes over 6 repetitions of the method (2.8° in FE axis, 1.2° in PS axis). However, the movement used to generate the angular velocity vectors was carefully constrained with the help of a bar and a table for support. In [19], a similar *functional* method was used to estimate FE and PS axes, with a larger cohort (15) and no supporting props, which resulted in RMSD values of approximately 11° and 13.5° for the FE and PS joint angles, respectively. In our study, the joint axis estimation algorithm was evaluated with 20 subjects, with no supporting props to constrain movement. We tested the algorithm using either “isolated” or “functional” movement and found that it performed best when “isolated” movement was used, where the average difference from the reference FE and PS axes was 8.9° and 6.3°, respectively (from 5 repetitions). It is important to note that, although we have labelled it the “isolated” movement trial, the movements were not, in reality, perfectly uniaxial. In fact, during the FE movements, the participants pronated/supinated through a range of 22° on average, and during the PS movement, elbow flexion/extension moved through 16° on average. This demonstrates that the algorithm was not relying on perfect isolation of each joint axis during the calibration movement. Therefore, this approach shows potential for calibration using motion which is not perfectly constrained, in comparison to the existing *functional* calibration methods.

Despite this, the success of the joint axis estimation algorithm *was* significantly worse when certain periods of “functional” movement were used. This could potentially be attributed to increased STAs when the upper arm is lifted away from the side of the thorax, causing the relative movement of the sensors to deviate from the 2 DoF joint model. Alternatively, insufficient range or variation in both FE and PS motion may have an adverse impact, demonstrated by the poor results using only the “kettle” or “drinking” task. The exact requirements of the calibration movement are yet to be identified and there is a risk that patients with severe motor impairments may not be able to achieve the required movement, therefore, for clinical application, validation with the target patient group would be necessary to ensure an accurate calibration could be achieved. Additionally, it should be noted that the proposed method also depends on a single static pose to calibrate the thorax sensor, therefore, it should be verified that the target population is able to achieve an upright posture, to some degree of accuracy.

In terms of *manual unit alignment*, shoulder and elbow joint RMSEs of 4.4°–6.5° were achieved in one study which included 8 subjects and 1 operator [20]. However, without characterising variability between a large range of subjects and operators, we cannot have confidence that this approach would be reliable in a clinical setting. In this study, the attachment mounts were proposed to provide a more repeatable manual alignment, with less dependence on operator time and expertise. On a cohort of twenty participants with a large variation in weight (51–126 kg) (min-max) and height (153–194 cm) and an equal number of females and males, the design was shown to align the sensors with an average error of 8.4° for the humerus bone’s y-axis, and 5.1° for the radius bone’s x-axis. The basic manufacture of the mounts makes them easily reproducible, and future work could investigate the implications of various design features, and whether multiple size options might provide better alignment. One aspect of the alignment which can not be characterised from this experimental trial is the dependence on the operator’s technique when attaching the mounts. The operator tried to emulate the actions of a non-technical clinician who had been given some basic directions, and placed the mounts as described in Section II-A3, then tightened the elastic, allowing the mounts to self-align. However, underlying knowledge of the implications of the placement may have subliminally affected the operator’s placement of the mounts, therefore, further testing should evaluate inter and intra-operator reliance, with clinicians acting as the operators.

Combining the approaches described above into a novel sensor-to-segment calibration method, joint kinematics were calculated and compared to those derived from the OMC measurements. The proposed method, using the “isolated” movement for calibration, resulted in shoulder and elbow joint angles with median RMSEs ranging from 5.5° in elbow flexion angle, to the highest errors of 8.1° in forearm pronation. When a period of “functional” movement was used in the calibration, the kinematic errors were higher for all joint angles, with median RMSEs ranging from 7.3° for shoulder flexion to 9.9° for elbow flexion. The highest errors in the forearm pronation angle likely derive from the aspects of the calibration which were particularly unreliable, with respect to both the forearm and humerus calibration. For example, calibration of the forearm’s orientation around the long axis was primarily dependent on the alignment of the forearm mount, and the average misalignment of the forearm mount on the ZX plane was 4.7° (5.1°), illustrated in Figure 5b). Additionally, the relative orientation of the humerus was defined largely by the estimate of the FE axis, which showed the largest variation when investigating the axis estimation algorithm. Other than the quality of the calibration, these joint angle errors could also stem from an accumulation of other factors, such as the inverse kinematics solver, the model used in OpenSim, or the sensor orientation estimates themselves. Moreover, these “errors” are, in fact, differences from the OMC system measurements, which also have inherent errors relative to the ground truth.

A comparison can be made to another study which has proposed an upper limb calibration method for clinical application: in [3], Picerno et al. used a sensor-mounted calliper to measure the direction of bone segment coordinate frames based on palpable bony landmarks, and achieved much lower median RMSD values (1.8°–4.4°). Compared to the method proposed in this study, calliper-based calibration relies more heavily on the actions of the operator, and is likely to be more time-consuming, although this has not been explicitly tested. Nevertheless, the key advantage of the calliper method is that it is entirely independent of the subject’s ability to maintain a pose or perform a motion, making it particularly suitable for patients with severe motor impairments, even if they are bedridden.

Considering the potential end-use of this technology, the errors associated with our *model-based* calibration will be acceptable for some applications, but unacceptable for others. For example, a standard set by the Clinical Movement Analysis Society - UK and Ireland for gait analysis laboratories advises a threshold error of 5–10° for inter-operator repeatability [21]. If similar standards are required from IMC measurements, then the errors associated with the proposed method may be deemed unacceptable, since some subjects had RMSEs higher than 10°. However, for other clinical applications, the tolerance for measurement error will be higher, depending on the minimal difference which impacts clinical decision-making. For example, for tracking joint range of motion progress during physiotherapy treatment, RMSE errors up to 10–20° may be acceptable.

This study also made a comparison between the proposed calibration method and the default calibration option built in to the OpenSense workflow, which relies on a static pose. We found that the static pose calibration resulted in larger median RMSEs for all joint angles, ranging from 6.2° to 11.5°, even when the operator provided assistance to achieve the pose. Although the errors are not significantly higher than those of the proposed method, it should be noted that these are the results associated with 20 *healthy* participants, and so errors would likely be higher with patients with limited movement control, or if the pose was performed at home, without supervision from a clinician. This result demonstrates the impracticality of a static pose calibration for clinical settings, when time is often limited and the patients may have even less control of their movement than the participants in this trial. In [22], Robert-Lachaine et al. also tested the success of static pose calibration with 12 healthy participants, and found that the N-pose resulted in an average misalignment of 12.5° and 9.0° in the longitudinal axes of the forearm and humerus, respectively. They also found that a chair could be used to achieve more reliable pose execution, but the use of custom arm mounts adjusted for each individual limits the clinical practicality of this approach.

A potential limitation of the model-based method described in this work is that it is intended for use with nine-axis inertial sensors which use magnetometer data to define their relative heading. The original approach described by Laidig et al. was proposed so that six-axis sensors could be used, avoiding the dependence on magnetometer data. Magnetic interference is a common issue in IMC, and was avoided in this study simply by avoiding proximity to potential magnetic disturbances, but this may not be possible in real-world applications. Therefore, although this study has demonstrated the effectiveness of the proposed calibration method in “clean” magnetic environments, further development of the six-axis axis estimation algorithm may be required before real-world implementation. Alternatively, the use of nine-axis sensors may become feasible with the development of nine-axis sensor fusion algorithms, such as VQF [23] which uses a magnetic disturbance rejection feature.

Another limitation of the proposed system is the use of elastic straps instead of adhesive tape, increasing the risk of slippage during movement, which compromises the calibration, and would particularly impact the proposed method as it partially relies on mount alignment. However, a major advantage of this method is that re-calibration can be performed quickly and easily once the mounts are re-tightened. Similarly, the use of arbitrary motion for calibration offers the potential for regular, periodic re-calibration, provided a suitable movement period can be automatically identified.

If the calibration method described in this work can be validated for use with clinicians and patients, further development could include implementation of the calibration in real-time, as demonstrated in [15], where automatic re-calibration could be used to periodically update the calibration from arbitrary motion. A calibration like this could make IMC significantly more accessible for clinical application, providing clinicians and patients with quantitative measures or 3D visualisations of their motion.

## V. Conclusion

This work has described a sensor-to-segment calibration method which, from only a short period of elbow motion, can be used to measure shoulder and elbow joint kinematics with median RMSEs of 5–8°. The proposed method is very quick to perform (5–10 s) and does not require precise poses or motions, making it a practical option for inertial motion capture in a clinical environment. The development of such a simple protocol is expected to increase the likelihood of the adoption of IMC as a clinical tool, and ultimately facilitate the translation of this technology into clinical practice. Further validation work should develop real-time implementation, investigate sensitivity to magnetic interference, and involve clinicians and patients for end-user feedback.

## Supplementary Materials

Supplementary Materials
