# Supplementary material for: A Practical Sensor-to-Segment Calibration Method for Upper Limb Inertial Motion Capture in a Clinical Setting
Source: IEEE J Transl Eng Health Med. 2025 Apr 30;13:216–26. doi: 10.1109/JTEHM.2025.3565986 (PMC12250904; doi:10.1109/JTEHM.2025.3565986)
Supplement: Supplementary Materials [file supp1-3565986.pdf]

Appendix A - Model Definitions

TABLE I: Definition of Anatomical Coordinate Frame with Proposed Calibration Method

<table>
<tr><th>Bone Segment</th><th>Label</th><th>ACS</th><th>Identification Method</th></tr>
<tr><td rowspan="3">Thorax</td><td>Yt</td><td>Proximal-distal</td><td>Coincident with $-\boldsymbol{g}$ at moment of static calibration pose</td></tr>
<tr><td>Xt</td><td>Medio-lateral</td><td>Common line perpendicular to thorax sensor's Z_{S_t} and Y_{S_t} axis, pointing to the right</td></tr>
<tr><td>Zt</td><td>Antero-posterior</td><td>Common line perpendicular to X_{S_t} and Y_{S_t}, pointing backwards</td></tr>
<tr><td>Humerus</td><td colspan="3">Orientation of humerus frame (B_h) relative to humerus sensor frame (S_h) is calculated in one step, solving Wahba's problem, to best align the vector pair $[\boldsymbol{j}_{FE}]_{S_h}$ with $[\boldsymbol{j}_{FE}]_{B_h}$ and the vector pair $[\boldsymbol{v}_y]_{S_h}$ with $[\boldsymbol{v}_y]_{B_h}$.

$[\boldsymbol{j}_{FE}]_{S_h}$ is the functional FE axis estimated from the relative angular velocities of S_h and S_r, and $[\boldsymbol{j}_{FE}]_{B_h}$ is the direction of the FE axis relative to the humerus bone, defined by the model used in the analysis. $[\boldsymbol{v}_y]_{S_h}$ is the y axis of the humerus sensor, and $[\boldsymbol{v}_y]_{B_h}$ is the y axis of the humerus body.</td></tr>
<tr><td>Radius</td><td colspan="3">Orientation of radius frame (B_r) relative to radius sensor frame (S_r) is calculated in one step, solving Wahba's problem, to best align the vector pair $[\boldsymbol{j}_{PS}]_{S_r}$ with $[\boldsymbol{j}_{PS}]_{B_r}$ and the vector pair $[\boldsymbol{v}_x]_{S_r}$ with $[\boldsymbol{v}_x]_{B_r}$.

$[\boldsymbol{j}_{PS}]_{S_r}$ is the functional PS axis estimated from the relative angular velocities of S_h and S_r, and $[\boldsymbol{j}_{PS}]_{B_r}$ is the direction of the PS axis relative to the radius bone, defined by the model used in the analysis. $[\boldsymbol{v}_x]_{S_r}$ is the x axis of the radius sensor, and $[\boldsymbol{v}_x]_{B_r}$ is the x axis of the radius body.</td></tr>
</table>

TABLE II: Definition of Joint Coordinate System of OpenSim Model

<table>
<tr><th>Joint</th><th>DoFs</th><th colspan="2">JCS</th><th>Zero Joint Configuration</th></tr>
<tr><td rowspan="3">Humerothoracic</td><td rowspan="3">3</td><td>Flexion/Extension</td><td>Projected vector of humerus z-axis on the thorax yz plane</td><td>Humerus z-axis coincident with thorax z-axis</td></tr>
<tr><td>Abd/Adduction</td><td>Projected vector of humerus y-axis on the thorax xy plane</td><td>Humerus y-axis coincident with thorax y-axis</td></tr>
<tr><td>Internal/External Rotation</td><td>Projected vector of humerus x-axis on the thorax xz plane</td><td>Humerus x-axis coincident with thorax x-axis</td></tr>
<tr><td rowspan="2">Elbow</td><td rowspan="2">2</td><td>Flexion/Extension</td><td>Rotation of ulna around OpenSim model's FE axis, which is fixed in humerus frame</td><td>Ulna z-axis aligned with humerus z-axis</td></tr>
<tr><td>Pronation/Supination</td><td>Rotation of radius around OpenSim model's PS axis, which is fixed in ulna frame</td><td>Radius z-axis aligned with ulna z-axis</td></tr>
</table>
