# Supplementary material for: A Practical Sensor-to-Segment Calibration Method for Upper Limb Inertial Motion Capture in a Clinical Setting
Source: IEEE J Transl Eng Health Med. 2025 Apr 30;13:216–26. doi: 10.1109/JTEHM.2025.3565986 (PMC12250904; doi:10.1109/JTEHM.2025.3565986)
Supplement: Supplementary Materials [file supp2-3565986.pdf]

Appendix B - Additional Results

TABLE I: Mean ROM of Each Joint Angle During Tasks

**Task**	**ROM (°)**				
	**Elbow Flexion/Extension**	**Elbow Pronation/Supination**	**Shoulder Abduction**	**Shoulder Flexion**	**Shoulder Rotation**
1 rep of FE and PS	93.36	131.06	14.26	13.61	22.47
5 reps of FE and PS	99.41	134.13	16.92	18.18	27.62
5 reps of FE	99.41	21.77	8.71	14.35	25.77
5 reps of PS	16.14	134.13	11.14	7.95	14.24
Kettle	51.65	88.04	45.95	46.27	53.54
Drink	107.32	53.5	45.65	58.51	65.01
Both	109.4	94.54	55.1	64.36	74.05

TABLE II: Optimisation Algorithm Joint Axis Estimates - Difference Compared to Reference Vector

**Input Movement**	**Joint Axis**	**Difference (°)**		
		**Median (IQR)**	**Min**	**Max**
Isolated (5 reps)	FE	8.9 (8.9)	3.1	22.6
	PS	6.3 (3.5)	1.5	10.7
Isolated (1 rep)	FE	10.5 (9.1)	2.2	25.1
	PS	5.9 (3.0)	1.8	11.8
Functional (drink)	FE	16.9 (17.8)	4.5	45.8
	PS	16.6 (8.6)	1.9	41.7
Functional (kettle)	FE	34.4 (33.9)	6.4	90.4
	PS	10.4 (20.8)	0.6	51.6
Functional (both)	FE	12.7 (19.3)	3.0	40.7
	PS	8.3 (6.1)	2.4	31.8

TABLE III: Error in the Sensor Alignment Provided by the Attachment Mounts

**Sensor Axis**	**Misalignment (mean (SD)) (°)**			
Humerus Sensor y-axis	Total	8.4 (3.0)	On ZY Plane (Shoulder Flexion)	5.7 (3.2)
			On XY Plane (Shoulder Abduction)	5.2 (3.4)
			On ZX Plane (Shoulder Rotation)	10.0 (11.3)
Radius Sensor x-axis	Total	8.4 (3.0)	On ZX Plane (Forearm Pronation)	4.8 (5.1)
			On YX Plane (Elbow Flexion)	1.3 (1.6)
			On YZ Plane (Elbow Abduction)	2.1 (2.5)

TABLE IV: Accuracy of IMC Joint Kinematics from the Isolated Movements Trial

**Calibration Method**	**Joint Angle**	**RMSE (°)**			**R**		
		**Median (IQR)**	**Min**	**Max**	**Median (IQR)**	**Min**	**Max**
New Method (Isolated)	Elbow Flexion	5.5 (2.6)	3.6	12.1	0.99 (0.01)	0.97	1.00
	Forearm Pronation	8.1 (5.4)	3.6	24.3	0.98 (0.02)	0.92	0.99
	Shoulder Abduction	6.5 (5.4)	3.2	19.7	0.98 (0.02)	0.88	0.99
	Shoulder Flexion	6.3 (5.5)	2.0	15.2	0.99 (0.01)	0.96	1.00
	Shoulder Rotation	6.7 (5.7)	3.0	21.1	0.98 (0.01)	0.92	1.00
New Method (Functional)	Elbow Flexion	9.9 (9.1)	5.1	26.4	0.97 (0.02)	0.80	0.99
	Forearm Pronation	9.3 (4.4)	4.4	18.8	0.97 (0.03)	0.81	0.99
	Shoulder Abduction	9.7 (3.5)	3.8	21.6	0.95 (0.05)	0.77	0.99
	Shoulder Flexion	7.3 (5.7)	2.9	14.5	0.99 (0.01)	0.93	1.00
	Shoulder Rotation	8.5 (5.5)	3.5	32.1	0.97 (0.04)	0.86	0.99
Pose Calibration (Self-Executed)	Elbow Flexion	11.4 (9.1)	4.0	18.7	0.99 (0.02)	0.90	0.99
	Forearm Pronation	13.5 (14.3)	3.5	31.6	0.97 (0.03)	0.80	0.99
	Shoulder Abduction	11.0 (6.3)	3.0	32.0	0.97 (0.05)	0.78	0.99
	Shoulder Flexion	7.3 (4.6)	2.6	19.9	0.99 (0.01)	0.93	1.00
	Shoulder Rotation	10.8 (6.7)	3.6	21.5	0.93 (0.07)	0.70	0.99
Pose Calibration (Assisted)	Elbow Flexion	8.9 (4.0)	4.3	17.2	0.98 (0.02)	0.91	0.99
	Forearm Pronation	11.5 (7.1)	3.9	22.1	0.98 (0.02)	0.92	0.99
	Shoulder Abduction	10.9 (5.7)	3.8	27.6	0.95 (0.06)	0.82	0.99
	Shoulder Flexion	6.2 (6.5)	2.2	15.9	0.99 (0.02)	0.94	1.00
	Shoulder Rotation	10.0 (5.4)	4.8	22.7	0.95 (0.04)	0.68	0.98
